# Improving diagnosis for rare diseases: the experience of the Italian undiagnosed Rare diseases network

**DOI:** 10.1186/s13052-020-00883-8

**Published:** 2020-09-14

**Authors:** Marco Salvatore, Agata Polizzi, Maria Chiara De Stefano, Giovanna Floridia, Simone Baldovino, Dario Roccatello, Savino Sciascia, Elisa Menegatti, Giuseppe Remuzzi, Erica Daina, Paraskevas Iatropoulos, Bruno Bembi, Rosalia Maria Da Riol, Alessandra Ferlini, Marcella Neri, Giuseppe Novelli, Federica Sangiuolo, Francesco Brancati, Domenica Taruscio

**Affiliations:** 1 National Centre for Rare Diseases, Undiagnosed Rare Diseases Interdepartmental Unit, Istituto Superiore di Sanità, Rome, Italy; 2 Department of Educational Science, University of Catania, Catania, Italy; 3 National Transplantation Centre, Istituto Superiore di Sanità, Rome, Italy; 4 Bioethics Unit, Istituto Superiore di Sanità, Rome, Italy; 5 Department of Clinical and Biological Sciences, University of Turin and S. Giovanni Bosco Hospital, Centre of Research of Immunopathology and Rare Diseases – Regional Coordinating Centre of the National Network for Rare Diseases, Turin, Italy; 6 IRCCS Mario Negri Pharmacological Research Institute, Regional Coordinating Centre of the National Network for Rare Diseases, Clinical Research Centre for Rare Diseases “Aldo e Cele Daccò”, Ranica, Bergamo, Italy; 7 S.O.C. Regional Coordinating Centre of the National Network for Rare Diseases, S. Maria della Misericordia Hospital, Udine, Italy; 8 Department of Experimental and Diagnostic Medicine, University of Ferrara, Ferrara, Italy; 9 Department of Biomedicine and Prevention, University of Tor Vergata and University Hospital Tor Vergata, Unit of Medical Genetics Rome & IRCCS Neuromed, Pozzilli, Italy; 10 Department of Biomedicine and Prevention, University of Tor Vergata and University Hospital Tor Vergata, Unit of Medical Genetics, Rome, Italy; 11 Department of Life, Health and Environmental Sciences, Unit of Medical Genetics University of L’Aquila, L’Aquila, Italy

**Keywords:** Undiagnosed, Rare diseases, Omics, Ontology, Diagnosis

## Abstract

**Background:**

For a number of persons with rare diseases (RDs) a definite diagnosis remains undiscovered with relevant physical, psychological and social consequences. Undiagnosed RDs (URDs) require other than specialised clinical centres, outstanding molecular investigations, common protocols and dedicated actions at national and international levels; thus, many “Undiagnosed RDs programs” have been gradually developed on the grounds of a well-structured multidisciplinary approach.

**Methods:**

The Italian Undiagnosed Rare Diseases Network (IURDN) was established in 2016 to improve the level of diagnosis of persons with URD living in Italy. Six Italian Centres of Expertise represented the network. The National Centre for Rare Diseases at the Istituto Superiore di Sanità coordinates the whole project. The software PhenoTips was used to collect the information of the clinical cases.

**Results:**

One hundred and ten cases were analysed between March 2016 and June 2019. The age of onset of the diseases ranged from prenatal age to 51 years. Conditions were predominantly sporadic; almost all patients had multiple organs involvements. A total of 13/71 family cases were characterized by WES; in some families more than one individual was affected, so leading to 20/71 individuals investigated. Disease causing variants were identified in two cases and were associated to previously undescribed phenotypes. In 5 cases, new candidate genes were identified, although confirmatory tests are pending. In three families, investigations were not completed due to the scarce compliance of members and molecular investigations were temporary suspended. Finally, three cases (one familial) remain still unsolved. Twelve undiagnosed clinical cases were then selected to be shared at International level through PhenomeCentral in accordance to the UDNI statement.

**Conclusions:**

Our results showed a molecular diagnostic yield of 53,8%; this value is comparable to the diagnostic rates reported in other international studies. Cases collected were also pooled with those collected by UDNI International Network. This represents a unique example of global initiative aimed at sharing and validating knowledge and experience in this field. IURDN is a multidisciplinary and useful initiative linking National and International efforts aimed at making timely and appropriate diagnoses in RD patients who still do not have a confirmed diagnosis even after a long time.

## Background

Rare diseases (RDs) are conditions affecting less than 5 individuals in 10.000 in Europe and fewer than 200.000 people in the US [1, 2]. Despite their low prevalence, RDs are collectively common conditions involving 6 to 10% of the population, raising critical issues for the whole community in terms of best clinical practices, diagnosis achievability, health services planning and public health programs. The exact number of RDs remains unknown: estimates approaching and exceeding 8000 conditions have been expressed [3, 4]. A substantial number of RDs are due to altered functions of single genes, whereas only a small proportion of RDs recognizes non-genetic causative factors. In these cases, documentation of environmental exposure, humoral biomarkers and drugs assumption alongside with the identification of risk factors is pivotal to support actions for primary prevention (i.e maternal levels of folic acid and neural tube defects, prenatal exposure to antiepileptic drugs, etc) [5].

RDs are often complex conditions requiring integrated, long-term care delivery settings and highly specially designed management, for whom due to the prevailing onset in childhood, paediatric specialists are almost unavoidable involved. Though their chronic and often progressive course, long-term complications of RDs can be lessened or delayed by early diagnosis; optimal management and prompt supportive and/or targeted therapies where available. In addition, appropriate and timely diagnosis ameliorate patient health status reducing psychological and social burden of the diseases and allow proper genetic counselling [6]. In the last decade, substantial effort has been made by the scientific community to increase the diagnostic rate of RDs through advanced molecular investigations. This allowed a better understanding of the molecular aspects of RDs, supporting diagnosis, clinical decision-making, more suitable choices of treatment and likely reducing financial costs.

Even so, for approximately half of persons with RDs, a definite diagnosis remains undiscovered [7]. Thus, for this substantial number of patients and their families, physical, psychological and social consequences represent a relevant burden to be sustained every day. In this respect the unsolved cases, formally defined as undiagnosed RDs (URDs), represent a tremendous additional hurdle, requiring to be solved other than extremely specialised clinical centres, outstanding molecular investigations, common protocols, dedicated actions at national and international levels such as the development of national “Undiagnosed RDs programs”. These programs have advanced operational procedures based on an individualized, exhaustive and well-structured approach to URDs case by a multidisciplinary team of experts.

In 2008, the National Institutes of Health (NIH) of USA launched first the Undiagnosed Diseases Program (UDP) to address an unmet need in the US health care system: the diagnosis of unsolved RDs cases [8, 9]. In 2014, inspired by the USA UDP, the Undiagnosed Diseases Network International (UDNI, http://www.udninternational.org/) was founded by five participants countries including Italy [10–12]. The National Centre for Rare Diseases (NCRDs) – Istituto Superiore di Sanità (ISS) (NCRDs-ISS) was the Italian representative in this network. Following and paralleling to other European experiences, in 2016 the Italian Undiagnosed Rare Diseases Network (IURDN) was launched within a bilateral project Italy-USA, through the Italian Ministry of Foreign Affairs and International Cooperation funds [13, 14]. The IURDN aimed to offer to patients suffering from URDs additional chances to get a definitive diagnosis by means of a large community of experts mostly belonging to the National Network of Clinical Centres for RDs, working together in a globally coordinated framework [15]. Therefore, main objectives of the IURDN were to enhance the rate of diagnosis in previously undiagnosed individuals to address subsequent clinical decisions, improve health care quality, identify new causes of diseases and foster cohesion, collaboration and meaningful connections across national and international organizations in the fields of URDs.

Here we describe the experience of IURDN, the analytical strategy and the results obtained in a 3-year-project, highlighting understanding of benefits, limitation and success factors of such initiatives in clinical practice.

## Methods

### The Italian undiagnosed Rare diseases network

**Fig. 1:**
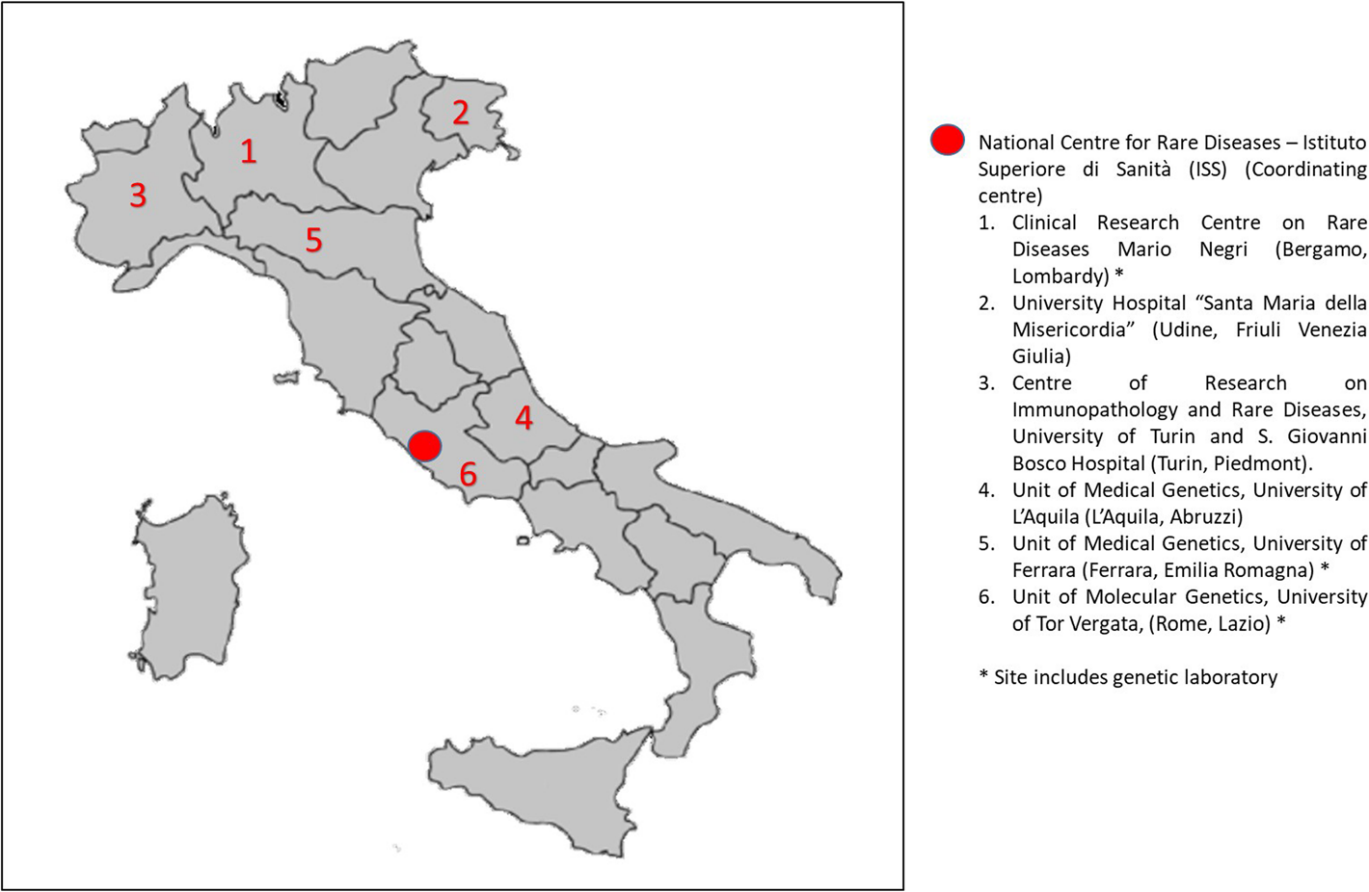
Geographical distribution of the 6 Italian Centres of Expertise for RDs involved in IURDN

The IURDN was established in 2016 as central part of the three-years project on “Undiagnosed Rare Diseases: a joint Italy – USA project” (PGR00229, 2016–19), funded by the Italian Ministry of Foreign Affairs and International Cooperation. The aim was to improve the level of diagnosis and optimise the management of persons with undiagnosed conditions living in Italy. The regulatory principles of IURDN (including role and functions of participants, criteria for patient’s selection, data collection and general work-flow) were clearly defined according to a detailed protocol previously published [13]. The network was organised as a non-hierarchically collaborative structure formed by independent partners, represented by 6 Italian Centres of Expertise for RDs (Fig. 1). Each partner had autonomous management of its own clinical cases, though each other were mutually dependant in achieving the common goals of the project. The National Centre for Rare Diseases (NCRDs) – Istituto Superiore di Sanità (ISS) (NCRDs-ISS) as coordinating centre of the whole project, was responsible for: a) coordination of the program; b) network governance, c) central management of the database Phenotips used for data collection, d) international data sharing; e) dissemination of results through a dedicated web site [14]. All six clinical sites included in the Network were Italian Centres of Expertise for RDs from the North and Central Regions of Italy.

Three of them were centres coordinating the Regional Network for RDs (according to the law/ACT NO. 279 of 2001 and subsequent update of the DPCM January 2017): 1) Clinical Research Centre on Rare Diseases Mario Negri (Bergamo, Lombardy); 2) University Hospital “Santa Maria della Misericordia” (Udine, Friuli Venezia Giulia); 3) Centre of Research on Immunopathology and Rare Diseases, University of Turin and S. Giovanni Bosco Hospital (Turin, Piedmont). The other three Centres were: 4) Unit of Medical Genetics, University of L’Aquila (L’Aquila, Abruzzi); 5) Unit of Medical Genetics, University of Ferrara (Ferrara, Emilia Romagna); 6) Unit of Molecular Genetics, University of Tor Vergata, (Rome, Lazio) [13, 14] (Fig. 1).

To warrant the intended heterogeneity of unsolved cases in term of reasons for referral, age at onset and clinical manifestations and in view of a cross-disciplinary approach, the participating centres had purposely-different expertise; three sites also included a laboratory core for genetic/genomic investigations (Lombardy, Emilia Romagna and Lazio).

### Patients/clinical cases recruitments

Clinical cases were recruited from those evaluated in one of the six Italian Centres of Expertise for RDs participating to the network. Each site was responsible for the identification of a number of the most suitable own undiagnosed cases among those referred for diagnostic purpose to their out-patients and in-patients clinics.

Clinical cases were presented to the board composed by clinicians, geneticists and basic scientists representative of the NCRD-ISS and of the six clinical centers in face-to-face meeting regularly organized in Rome at the NCRD-ISS. Periodical virtual brainstorming sessions were also organized to talk over the cases-study. Cases were selected according to inclusion criteria (see above), analyzed and discussed by the board in keeping with a stepwise procedure, as follows:

1. Plenary discussion to see whether a joint comprehensive re-evaluation of clinical and ancillary data could further contribute to get a definitive diagnosis;

2. Suggestion of possible diagnosis to be excluded by means of specialized genetic/biochemical laboratory tests and/or instrumental investigations not yet/previously performed;

3. For those cases remaining still undiagnosed, requested data were entered into the database *PhenoTipsISS* (see above);

4. Whole exome sequencing (WES) was considered and the convenience discussed;

5. Elect cases were addressed to functional and further studies;

6. Identification/collection of a number of undiagnosed cases to be shared at the international level through the PhenomeCentral repository (see above).

**Fig. 2:**
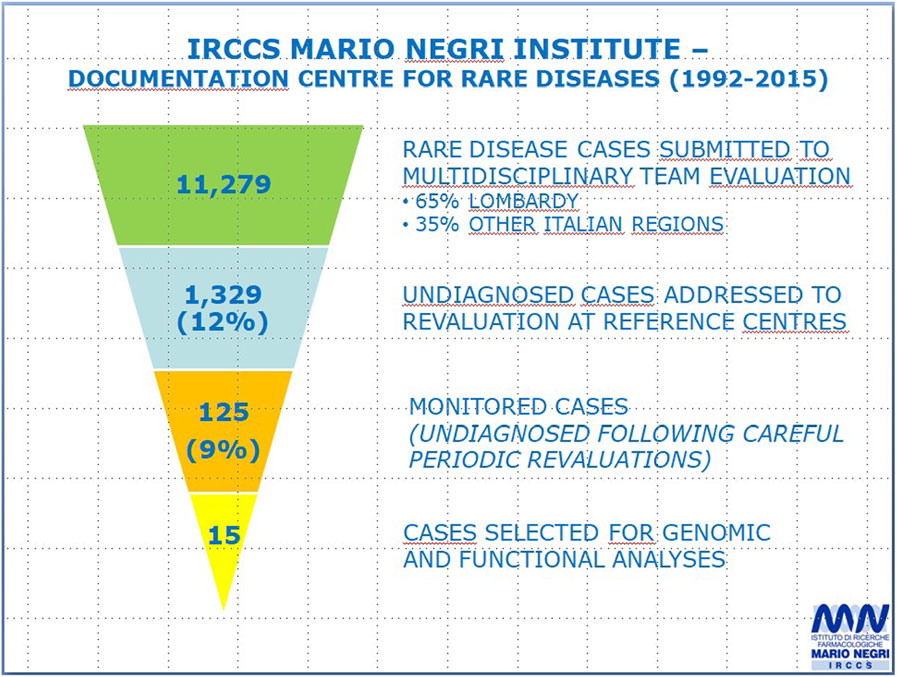
Process of selection of undiagnosed clinical cases at the Clinical Research Centre on Rare Diseases Mario Negri (Bergamo, Lombardy)

Figure 2 shows an example of the process of recruitment and inclusion of unsolved clinical cases in the IURDN; the example is referred to Clinical Research Centre on Rare Diseases Mario Negri (Bergamo, Lombardy) in the role of coordinating Centre of the Regional rare diseases Network in Lombardy (according to the law/ACT NO. 279 of 2001 and subsequent update of the DPCM January 2017). In 1992–2015 period a total of 11.279 referred patients with RDs were documented: 65% of them were from Lombardy and 35% were from other Italian regions. Twelve percent of the total number (1329 out of 11.279) had not a definitive diagnosis and were referred to experts of other Regional Centres for second or further opinions. Among the 11.279 patients, 125 cases (9%) were ultimately defined and monitored as undiagnosed cases and underwent to careful periodic revaluation. Only 15 out of 125 patients (12%) were selected by the centre to be presented and further discussed to IURDN. As the Centre boasts an excellent clinical and research experience in renal diseases of adulthood, selected unsolved cases referred to that.

### Inclusion criteria

Patients were selected for the unique characteristic of their undiagnosed disorder and for the potential to reveal new aspects of cell biology or biochemistry of their condition. Candidate cases had clinical signs and symptoms not yet clearly identifiable or attributable to a known RD, despite extensive clinical investigations and visits by a number of specialists for a long time (diagnosis still eluded).

The inclusion criteria, as stated elsewhere [13, 14], were the following: *a*) absence of a clear clinical diagnosis although extensively and thoroughly investigations; *b*) absence of a definitive molecular diagnosis though clinical manifestations supported a well-known diagnosis (i.e. no hypothesis/demonstration of cause-effect relationship over known genotype-phenotype correlation); *c*) either paediatric or adult patients; *d*) familiar or sporadic cases, ethnic isolates.

The minimum data set related to previous comprehensive assessment of any undiagnosed patient includes full clinical evaluation and diagnostic studies:

i) Biochemical (i.e. enzymes, ions, antibodies);
ii) Instrumental (i.e. ultrasound, NCS, MRI, neuropsychological tests);
iii) Biological samples: (i.e. biopsy);
iv) Genetic: (i.e. karyotype, chromosomal microarray analysis, targeted single-gene, gene-panel sequencing).

### ISS patients database - PhenoTipsISS

In order to collect and analyse the phenotypic information of the selected clinical cases, the software application “*PhenoTips*” [16] was used and customised for the purpose of the project (*PhenoTipsISS)*. This web-based application granted a standardized and indexable way of data collection, measurements and growth curves, diagnosis guidance based on the entered data, enabling also the interoperability among other databases, which incorporate human phenotypic features. Moreover, the Human Phenotype Ontology (HPO) vocabulary used in *PhenoTipsISS* guaranteed a **standardised phenotyping** [17, 18]. This tool contains over 8000 terms representing individual phenotypic anomalies and all clinical entries in Online Mendelian Inheritance in Man (OMIM) have annotated with the terms of the HPO.

The access to *PhenoTipsISS* was restricted by key codes and authenticated participants were associated to a specific working group, whose name was assigned accordingly to participant affiliation. Users had two different roles: as viewers (view the patient data without the ability to modify them), and as contributors/data owners (contribution to implementation of data and modification of their own entered data). The UDN-ISS working group was the server administrator (manages the access rights and the advanced settings) and had full access to the whole data, together with the ability to modify or remove all of them.

First, each patient was entered in *PhenoTipsISS* in one of the ten categories established to comply with the needs of the project and with regard to the expertise of the Centres involved: connective tissue disorders, endocrine genetics, intellectual disabilities syndromes, metabolic disorders, motor neurons diseases, congenital multiple abnormalities syndromes, myopathies, renal diseases, skeletal dysplasias, systemic autoinflammatory syndromes.

Data collected in the database include: patient information (examen date, sex and indication for referral); family history and pedigree (paternal ethnicity and global mode of inheritance); prenatal and perinatal history (pregnancy history and perinatal complications); medical history (global age of onset); measurements and growth charts; clinical symptoms and physical findings; genotype information (including list of genes already characterized); copy number variants; diagnosis (including possible matching to disorders listed in OMIM). Further details on the PhenoTips software are available at https://phenotips.org/.

### International databases and PhenomeCentral

On the grounds of what accomplished in UDNI (http://www.udninternational.org/) [10, 11], one of the objectives of IURDN was also to share a minimum of 5 undiagnosed cases, through the International PhenomeCentral, a repository for clinicians and scientists working in the RDs community (https://www.phenomecentral.org) [19, 20]. For this purpose, a dedicated working group, named “UDN-ISS Italy”, consisting of representatives of the IURDN, was established. Cases were set as “*matchable*” and shared via UDNI to PhenomeCentral. The NCRD-ISS was the administrator; the contact point that enters cases and receives email notifications about matched patients of the multi-centre research consortium.

### Genomic analysis

Next generation sequencing techniques, mainly whole exome sequencing (WES), was performed by trio analysis (proband, father and mother) on the selected cases included in PhenoTips repository.

Exome capture and next-generation sequencing was carried out at Personal Genomics (http://www.personalgenomics.it/) on an Illumina HiSeq2000 (Illumina, San Diego, CA) platform and indexed libraries were subjected to paired-end (2 × 100 bp read length) sequencing-by-synthesis using fluorescent reversible terminators. Exome enrichment was conducted following the protocol for the SeqCap EZ Human Exome beads (Roche NimbleGen, Inc., Madison, WI, USA).

For all samples, DNAs were isolated from peripheral blood of the patient with standard protocols, and whole exome sequencing (WES) was performed on both normal parents or unaffected brothers and sisters. Sequence reads were mapped to the human reference genome assembly (GRCh37/hg19) using CLC Genomics Workbench™ software (CLC bio, Aarhus, Denmark).

Variants were called, filtered, and prioritized according to their pathogenicity scores obtained from the MutationTaster, CADD, and Polyphen-2 web interfaces. Furthermore, variants were cross-referenced with the Human Gene Mutation Database (HGMD, http://data.mch.mcgill.ca/phexdb), and genes known to be implicated in ciliopathy-related disorders were prioritized.

**Fig. 3:**
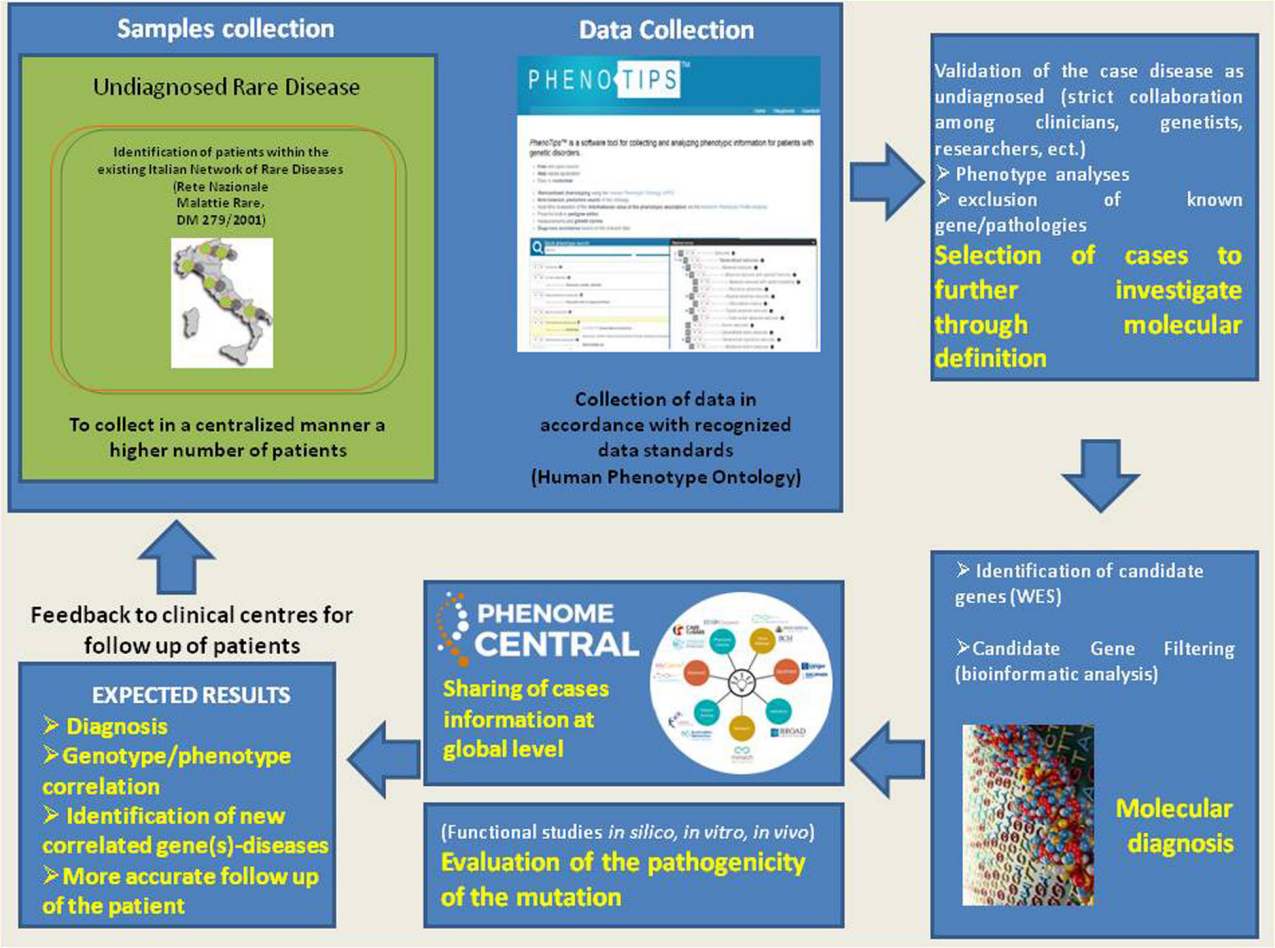
Whole process of progression (hierarchy) of the unsolved cases

In Family *P0000076* case, analyses were done by using the NimbleGen SeqCap EZ Human Exome Library v3.0 (Nimblegen 64 Mb) exome capture kit (NimbleGen, Madison, WI, USA) according to the manufacturer’s instructions as described previously [21] and enriched libraries were sequenced by Illumina Hiseq2000 platform (Illumina, Inc. San Diego, CA) with 90-bp paired-end reads. Sequencing reads were aligned to the human reference genome (NCBI build 37.1, hg19) using both SOAPaligner and BWA with default parameter. SNVs and Indels were detected by using SOAPsnp (soap2.21) [22] and GATK (version v1.0.4705) [23]. Polymorphic sites of detected variants were filtered by three public databases, including 1000 Genomes Project (http://www.1000genomes.org/), ESP (https://esp.gs.washington.edu/drupal/), ExAC (http://exac.broadinstitute.org/) with MAF > 0.5% and BGI-In-house database. Since parents were consanguineous, we used homozygosity mapping analysis [24] to first detect the homozygous region narrowing down the candidate gene list. Figure 3 summarises the whole process of progression (hierarchy) of the unsolved cases.

### Ethical issues

IURDN uses two different informed consents (IC) signed by patients and/or their relatives: one for the inclusion clinical case in to IURDN and the second to perform genomic analyses. Both IC were signed-up at the beginning of the study and were kept by the participating clinical centres.

## Results

A total number of 110 cases were collected and studied during the in-person meetings and the conference calls hold by IURDN, between March 2016 and June 2019.

Thirty-nine out of 110 cases (35,4%) were excluded as they did not meet the inclusion criteria. In no case, the board was able to reach a definitive diagnosis on the grounds of the existing clinical notes and investigations.

Seventy-one clinical cases out of 110 (64,6%) were finally entered into *PhenoTipsISS*, referring to 6 out of the 10 categories previously designated (Table 1). No cases were assigned either to metabolic disorders, myophaties, skeletal dysplasias or systemic autoinflammatory syndromes.

**Table 1:** Distribution of the 71 clinical cases according to the designated categories in *PhenoTipsISS*

**Categories**	**N° of cases entered in to** ***PhenoTipsISS***
Connective tissue disorders	7
Endocrine genetics	1
Syndromes with intellectual disabilities	34
Metabolic disorders	0
Motor neuron diseases	2
Syndromes with congenital multiple abnormalities	19
Myopathies	0
Renal diseases	8
Skeletal dysplasias	0
Systemic autoinflammatory syndromes	0
**Total number of cases**	**71**

**Fig. 4:**
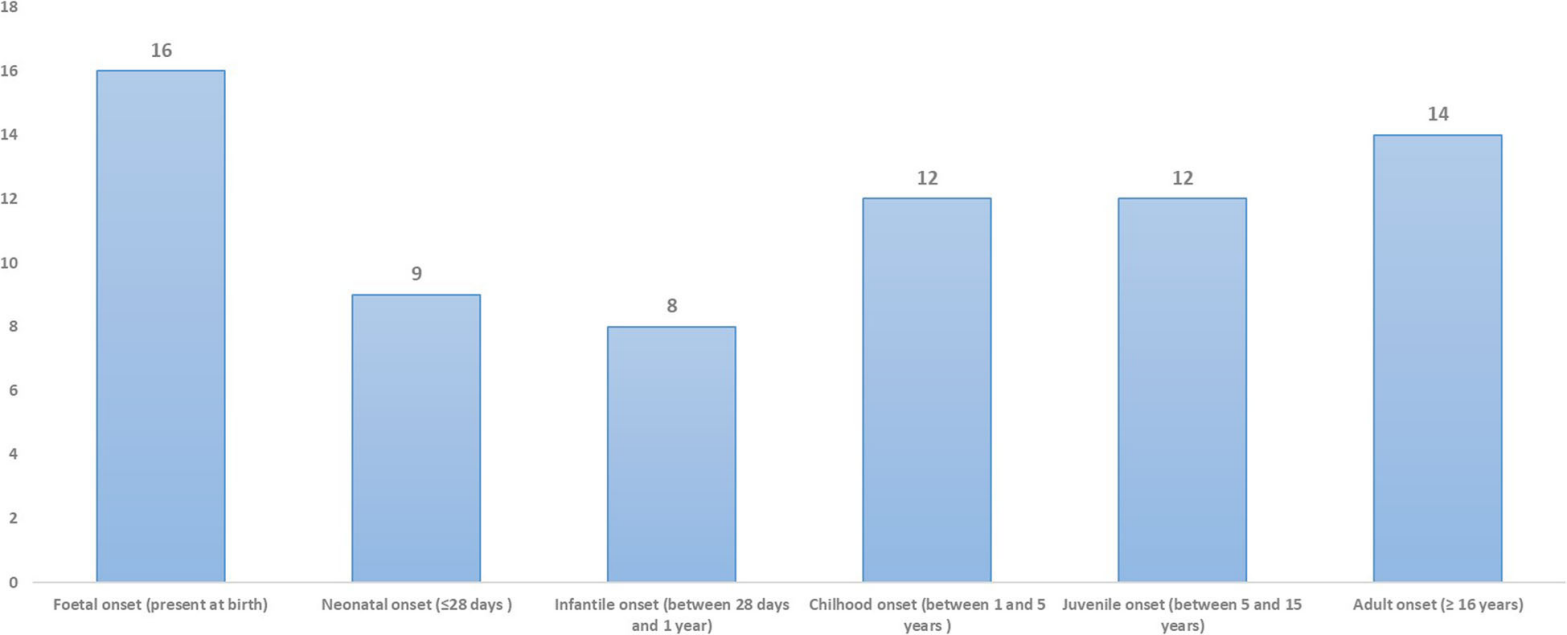
Onset class distribution according to PhenoTips® range. Distribution of the age at onset of clinical manifestations in the 71 cases entered in Phenotips. On Y axis, is reported the number of patients

Cases were distinct into different classes of age at onset fitting with the *categories expressed in PhenoTips: foetal, neonatal, infantile, childhood, juvenile and adult* (Fig. 4). The age of onset of the manifestations ranged from foetal period to 51 years. In 78.90% of cases (*n* = 56), the onset occurred in individuals younger than 16 years of age. Among these, 22.5% (*n* = 16) had phenotypic abnormalities present at birth, one case was detected during foetal life; 40,84% (*n* = 29) were aged from 1 month to 5 years; 16.90% (*n* = 12) were between the age of 5 and 15 years; 19,72% (*n* = 14) were older than 16 years.

The distribution of patients by gender was rather similar for both sexes (39 males and 32 females). However, this distribution showed a different pattern depending on the age of onset of signs and symptoms: a higher number of males (*n* = 31; 62%) compare to female (*n* = 18; 36%) was observed in patients with onset in younger age.

**Fig. 5:**
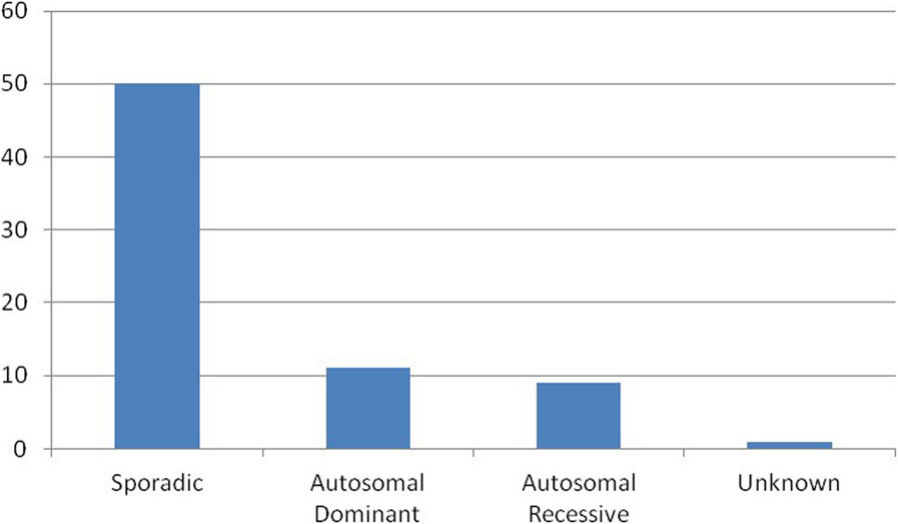
Cumulative mode of inheritance of the 71 cases included in PhenoTipsISS

Figure 5 shows the cumulative mode of inheritance of cases included in *PhenoTipsISS*. Conditions were predominantly sporadic (*N* = 50; 70,4%); 15,5% (*N* = 11) were autosomal dominant; 12,7% (*N* = 9) were autosomal recessive; 1,4% (*N* = 1) were unknown.

Overall, in almost all patients included in the database, the disease involved multiple organs; nervous system was, indeed, the most affected together with joint/skeletal and ocular systems. Four out of 71 (5,6%) had liver involvement only and in 2 additional patients systemic autoimmune and endocrine manifestations were present respectively. All cases were adult and not-related.

**Fig. 6:**
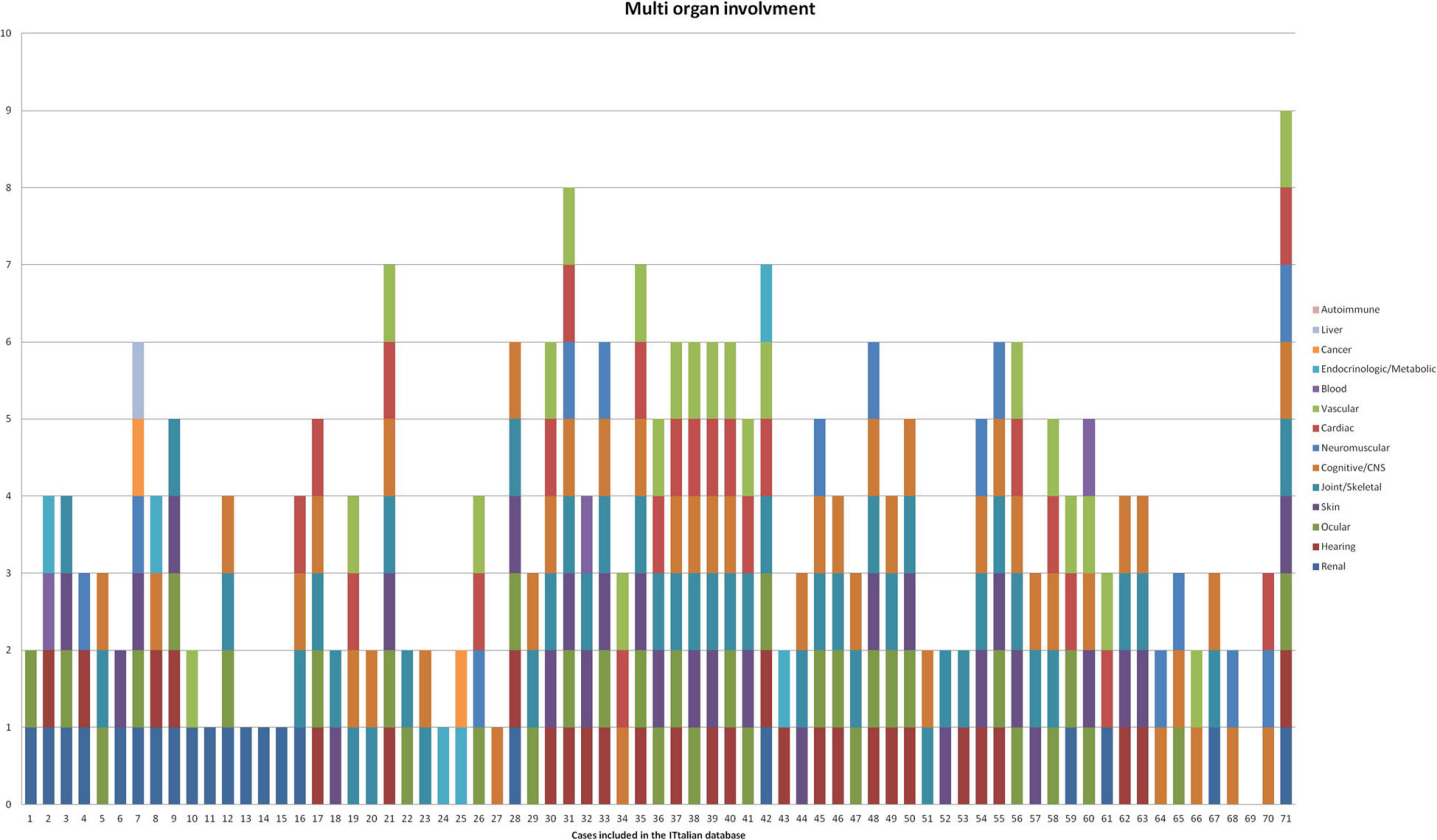
Combination of organs and/or systems involved in each of the 71 patients entered in *PhenoTipsISS*

Figure 6 summarises for each of the 71 undiagnosed patients, the affected organs and/or systems as entailed from clinical and instrumental evidence.

### Genomic analysis

A total of 13/71 (18,3%) family cases were selected to be characterized by WES, a few cases were familial, leading to a total number of 20 individuals investigated by WES (Table 2).

**Table 2:**
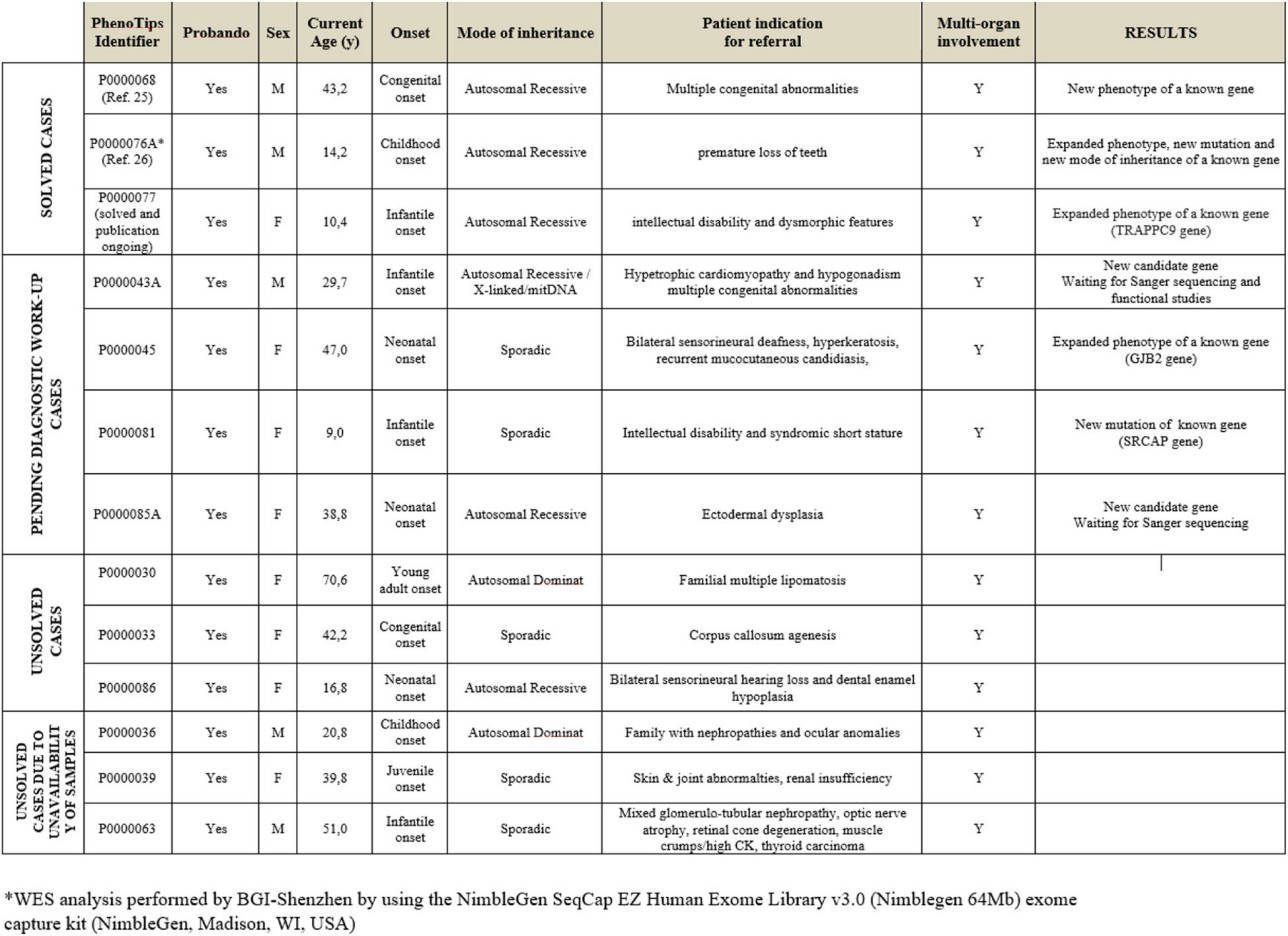
Description of cases selected to be characterized by WES.

Disease causing variants were identified in two cases (namely Phenotips P0000068 and P0000076A and B case identifiers) demonstrating homozygous and heterozygous variants associated with previously undescribed phenotypes [25, 26]. In 5 cases (namely Phenotips P0000043, P0000045, P0000077, P0000081, P0000085 A and B case identifiers), new candidate genes were identified, although confirmatory tests are still pending. In three family cases (namely Phenotips P0000036, P0000039, P0000063 case identifiers), investigations were not completed due to the scarce compliance of family members; for these cases, molecular investigations were temporary suspended due to the unavailability of the biological samples. Finally three cases (one familial) (namely Phenotips P0000030, P0000033 and P0000086 case identifiers) remain still unsolved (see Table 2).

### Description of solved cases

#### Case identifier in PhenoTipsISS: P0000068

WES was performed in the 38 yrs-old man affected by RYHNS syndrome (Retinitis pigmentosa, Hypopituitarism, Nephronophthisis and Skeletal dysplasia) previously described [27]. The analysis identified compound heterozygous variants in the ciliary gene *TMEM67*, each inherited from a heterozygous parent. Sanger sequencing confirmed a paternally inherited nonsense c.622A > T, p. (Arg208*) in exon 6 and a maternally inherited missense variant c.1289A > G, p.(Asp430Gly), near the splice acceptor site of exon 13, which perturbs the correct splicing of exon 13 of the gene*,* resulting in a c.[622A > T];[1289A > G] genotype. The missense variant was absent from the 1000 Genomes Project, the Exome Aggregation Consortium (ExAC, http://exac.broadinstitute.org) and the Genome Aggregation Database (gnomAD, http://gnomad.broadinstitute.org); conversely, the nonsense variant was present at extremely low frequency in the most comprehensive variant database with 49 out of 277,178 alleles reported in gnomAD.

*TMEM67* gene, encoding the transmembrane protein meckelin, is responsible of a group of diseases called human ciliopathies (REF). Overall, TMEM67 showed one of the widest clinical continuum observed in ciliopathies ranging from early lethality to adults with liver fibrosis. Our findings extend the spectrum of phenotypes/syndromes resulting from biallelic *TMEM67* variants to now eight distinguishable clinical conditions including RHYNS syndrome [25]. In this case, the molecular results were achieved by WES 20 years after the clinical diagnosis. Earlier detection would have allowed a tailored family-centred approach to genetic counselling to identify potential carrier, reduce health professional knowledge deficit, lessen the emotional distress of the family and the lingering worry about the second and third born infant health.

#### Case identifier in PhenoTipsISS: P0000076A e P0000076B

This was the case of two brothers from an inbred family of Pakistani origin affected by early tooth loss and absence formation of cementum. WES was performed on both the affected siblings and their normal parents (family-of-four strategy). Seventeen shared genes with homozygous variants in both affected brothers and heterozygous states in both normal parents were interrogated after only rare amino acid changing variants were kept. WES revealed a H2665L homozygous sequence variant in the VCAN gene (Chondroitin sulfate proteoglycan 2, versican or *CSPG2* gene) of both brothers.

Variations were prioritized based on the commonly shared genomic regions (inbreeding) and allele frequency (less than 0.5%). After the filtering process, the *VCAN* (Chondroitin sulfate proteoglycan 2, versican or *CSPG2)* gene was identified as the best candidate with a homozygous missense sequence variant in exon 8 (c.7994A > T; p.His2665Leu; chromosome 5: 82836816; GRCh37/hg19) (NM_004385.4). The homozygous sequence variant was present in both affected brothers and was heterozygous in parents. The variation MAF was = 0 in ExAc and 1000G databases, and it was only reported once in gnomAD^4^ in a heterozygous genotype not associated with a diseased status.

Sanger sequencing confirmed that finding. Dominant splicing mutations in VCAN are known to cause Wagner syndrome or vitreoretinopathy. Indeed, early signs of vitreoretinopathy were found in the older brother although his parents did not manifest any ocular involvement. This is the first case of peculiar developmental teeth anomaly related to a novel, homozygous, recessive, missense sequence variant in the *VCAN* gene. Thus, our findings expand both the phenotype mutation scenario and the mode of inheritance of VCAN mutations [26]. Again, the definitive diagnosis permitted the detection of early sign of vitreoretinopathy in a case, letting a combination therapy to target precocious stage of the disease and prevent further complication.

#### Case identifier in PhenoTipsISS: P0000077

The patient is a 10-yrs-old girl born from consanguineous parents from Morocco; the child had severe intellectual disability with a delayed speech and language development. She presented with dysmorphic features such as up-slanting palpebral fissures, thick lower vermilion lip and wide nasal bridge. Aggressive behaviour and stereotypic movements were also evident. WES analysis identified in the proband a homozygous mutation in TRAPPC9 gene (Trafficking Protein Particle Complex, Subunit 9) confirmed by Sanger sequencing. Nonsense mutations in TRAPPC9 have been reported in a total of 5 individuals with non-syndromic intellectual dysability from the Middle East, although brain abnormalities and obesity were described in only a few. In 2013, a pathogenic mutation in TRAPPC9 was found in two Italian sisters born to healthy unrelated parents [28]. Children had peculiar face, obesity, hypotonia, moderate to severe intellectual disability and brain abnormalities. A homozygous splice site mutation of TRAPPC9 causing exon skipping with frameshift and premature termination was detected. Considering the clinical and genetic heterogeneity of huge syndromes with intellectual impairment, the success of WES analysis is essential for attaining diagnosis in patients with a less peculiar phenotype such as the present.

### Description of cases with pending diagnostic work-up

#### Case identifier in PhenoTipsISS: P0000043A and P0000043B

Two brothers from apparently healthy unrelated parents, presented with peculiar facies, hypertrophic cardiomyopathy, scoliosis, pectus excavatum, hypergonadotropic hypogonadism and mild intellectual disability. In one brother, there was also a congenital heart defect of septal atrial ostium secundum type. All 4 family members underwent WES and results showed variants of uncertain significance; further investigations are under way.

#### Case identifier in PhenoTipsISS: P0000045

The proband was born at term following normal pregnancy and delivery. She is the second-born to non-consanguineous healthy parents. Soon after birth severe bilateral high frequency sensorineural deafness was diagnosed. At that age, maculo-papular eritematous lesions became also evident all over the trunk with sparing of the facial region. Long-standing chronic dermatitis evolved into keloid tissue. The girl also suffered from recurrent mucocutaneous candidiasis and premature loss of primary teeth. Lymphocytopenia with abnormal CD4/CD8 ratio was detected. Skin examination revealed spare bilateral eyelid, diffuse epidermal thickening, cheilitis and acne lesions. Marked plantar and palmar hyperkeratosis developed and progressed in severity thereafter. No constricting bands around fingers were noticed. A de novo mutation c.426C > A (p.Phe142Leu) of the gene GJB2 was found at exon sequencing. Functional studies are advancing.

#### Case identifier in PhenoTipsISS: P0000081

This 8-yrs-old girl is the second born to healthy unrelated parents. Her older brother is also healthy.

The perinatal history was unremarkable and anthropometric data at birth were within the normal range. At her past medical history there were recurrent episodes of lower respiratory and urinary tract infections. The child shows peculiar facies and postnatal proportionate short stature; her voice was abnormal; motor and language development were delayed; a hyperkinetic behaviour was also evident since infancy together with intellectual disability. Standard karyotype, array-CGH, FMR1 methylation, subtelomeric rearrangements and FISH *(11p15.5 and 8q24.11*) analysis were all negative.

The clinical picture was highly suggestive of the Floating-Harbor syndrome, Sanger sequencing/exon sequencing of exon 34 of the SRCAP gene, disclosed a de novo truncating mutation (c.7371delT), likely pathogenic. As in the previous case functional studies are in progress.

#### Case identifier in PhenoTipsISS: P0000085A and P0000085B

A sister and his affected brother were born to non – consanguineous Italian healthy parents. A third brother was also apparently health. Both sister and brother had ectodermal dysplasia with ankyloblepharon, dermatoglyphic, dysplastic nails, coarse hair, plantar hyperkeratosis. There were not hypohidrotic lesions. Hands and feet had bilateral syndactyly between the 2nd and 3rd fingers. Blood samples from all family members were not available; hence WES has been considered only for both the affected brothers. Autosomic homozygous and heterozygous variants commons in both brothers with an allelic frequency less or equal to 0.5 were evaluated: 20 and 150 different variants were found in homozygous and heterozygous conditions respectively. From this result a possible candidate gene was hypnotized and further analyses are currently under investigation.

### Description of unsolved cases

#### Case identifier in PhenoTipsISS: P00000030

This is a case of familial multiple lipomatosis, inherited as dominant trait. Probands are two sisters and one brother. Their deceased father and at least two paternal first cousins were also recognised to be affected by multiple lipomatosis.

WES was extended to all three probands and their unaffected mother, plus one affected paternal first cousin: a total of 4669 common variants were found among the individuals. Analysis of these variants was not conclusive for a diagnosis. An additional cousin has been included in the study and SNP-array analysis is currently ongoing to eventually identify regions in linkage and combine these results with WES ones.

#### Case identifier in PhenoTipsISS: P0000033

First born to healthy unrelated parents, she has motor and cognitive delay. Brain MRI showed partial agenesis of the corpus callosum and dilated lateral ventricles. WES analyses are actually ongoing.

#### Case identifier in PhenoTipsISS: P0000086

This is the case of a sister and her brother affected by amelogenesis imperfecta and bilateral sensorineural hearing loss. Parents are consanguineous. An older brother is healthy. WES analyses were performer on the proband and her healthy parents. Twenty-seven homozygous and 100 compound heterozygous variants were identified but their analysis was not conclusive. Analyses will be extended to healthy sister and affected brother.

### Cases shared at international level (UDNI)

Twelve undiagnosed clinical cases were then selected to be shared at International level through PhenomeCentral in accordance to the UDNI statement. This phase of the project enabled the cases to be matchable with other similar cases entered by the research community registered into the Web repository PhenomeCentral. Overall, 7 patients out of 12 were found to show potential matches with different relevance percentage of match similarities with other patients. In order to explore some further matches, additional comparisons are in progress between groups of researchers who uploaded the matching patient. Currently the matching process continues to take place as soon as new data entry are available.

### The dedicated website

A dedicated website (http://www.udnpitaly.com/eng-home) was also developed to better explain aims and finality of the project, spread participants activities and create an operative platform accessing to PhenoTipsISS for each clinical center involved in the Network [14].

## Discussion

To answer to the increasingly complex challenges RDs frequently face health authorities and the scientific community need to go beyond conventional policies and services. They need to design and experiment bold and innovative solutions and sustain advanced health programs in an era where genomic technology obviously drives the diagnostic process of many diseases. Thus, genomic medicine is currently used to investigate complex conditions, including suspected monogenic disorders, in prenatal, paediatric and adult age especially when referred to tertiary clinical centres [29–31].

Under-diagnosis and misdiagnosis represent a well-known burden for people with RDs and their families, also afflicted by anxiety and depression as experienced in about 40% of parents of undiagnosed children [32]. In this respect, Knott et al. report the struggle and exemplary experience of an Australian family in the search for a diagnosis for her baby girl affected by a variant of Rett syndrome [33]. Depicting the scenario of the long diagnostic journey that can occur elsewhere in the world for many unsolved complex conditions, the authors drive the attention to the high rate of cases remaining undiagnosed for many years likely due to the lack of specific screening tests and the limited knowledge of RDs. Clinicians should be aware of the range of possible clinical presentations and new variants of a given disorder when considering a diagnosis. Diagnostic delay is indeed a major concern representing a critical challenge for both patients and family quality of life [33, 34]. The utility of clinical exome sequencing in the diagnosis of critical ill infants suspected to have genetic disorders has been demonstrated in large samples of infants. The diagnostic yield is about 40% and demonstrated to positively influence the process of clinical decision making [29]. Many children and adults patients with unsolved conditions are eventually diagnosed as having rare conditions when the investigations are redirected to the genomic analysis [35]. The establishment of nation-wide well-organised plans for undiagnosed people centred on genomic technology could systematically improve quality of assistance, arrange tailored health services with a greater level of involvement in the care of such patients ensuring timely and appropriate diagnoses, suitable treatments and proper genetic counselling for their family.

Programs aimed at solving undiagnosed cases are currently being developed and growing quickly in many countries. In 2008, the National Institutes of Health (NIH) of USA launched the Undiagnosed Diseases Program (UDP) to improve the diagnostic rate of mysterious conditions [8, 9]. A prime mover in initiating this action was the consideration that for about one third of USA patients it took a few years to reach a proper diagnosis and more than 5 years for about 15% of them. Moreover, at least 6% of the inquiries to the Genetic and Rare Diseases Information Center were from individuals seeking a definitive diagnosis [8, 9]. A second critical motivation for establishing the program was to enhance the discovery of new insights into biochemistry, physiology and cell biology of known and novel diseases with relevant and/or undescribed clinical manifestations by joining up different expertises. In 2013, the Common Fund of the US NIH supported a nationwide Undiagnosed Diseases Network (UDN), established to bring together outstanding clinical and research centres across the United States to solve the most challenging medical mysteries using advanced technologies [8, 9]. In 2014, inspired by the US UDN, the Undiagnosed Diseases Network International (UDNI, http://www.udninternational.org/) was funded, five countries were originally involved: Austria, Bulgaria, Hungary, Italy and Sweden. Since then, a growing number of Nations joined to this program so that at the present Australia, Belgium, Canada, France, Germany, India, Israel, Japan, South Korea, Spain, Sri Lanka, The Netherlands, Thailand and the United States joined to the Network. Main aim of UDNI was to offer the chance of a definitive diagnosis to persons living with undiagnosed conditions, sharing clinical information and genomic data at international level [10–12]. Based on a consensus framework of principles and practices adopted by the participating members, UDNI represents a unique and blooming example of global initiative aimed at sharing and validating knowledge and experience on RDs in the world. Following all those pioneering experiences, other similar UDP initiatives have been launched in single European countries (Austria, Bulgaria, Hungary, Sweden, Italy and Spain) and in other part of the world (Japan, Korea, Australia, India and Canada) [10–12].

The Italian Undiagnosed Rare Diseases Network (IURDN) was established in 2016 within a bilateral project Italy-USA funded by the Italian Ministry of Foreign Affairs and International Cooperation. In this respect, being the NIH founder of several National (i.e. US Undiagnosed Disease Network) and International (Undiagnosed Diseases Network International) efforts and initiative on URDs, the Italian program could take advantage from such important know-how in terms of experience, protocols and standard operating procedures.

The project was coordinated by the National Center for Rare Diseases at the Istituto Superiore di Sanità (www.iss.it) in Rome. Main objectives were: *a*) to collect unsolved clinical cases through the Italian Network of Centres of Clinical Expertise for RDs (according to the law/ACT NO. 279 of 2001 and subsequent update of the DPCM January 2017); *b*) to develop a national database to facilitate data sharing at international level; *c*) to strengthen collaborations between Italy and USA by sharing phenotypic and genomic data, getting second opinions and achieving best practices [13]. Indeed, the IURDN was launched to offer to patients living in Italy and suffering from URDs additional chances to get a definitive diagnosis by means of valuable expert opinions and cutting-edge molecular technologies, within a globally coordinated framework [15].

In 3 years’ time, a total of 71 cases were selected and included into the IURPN database; the majority of unsolved cases were children aged between 0 and 5 years. This was not surprising as the onset of RDs usually occurs during the first years of life [34]. Data are also in line with what evidenced in the reports of the National Institutes of Health Undiagnosed Diseases Program where two peaks of age of applicants were relevant: one between 4 and 6 yrs. for conditions detected at birth and the second one between 16 and 18 yrs. for diseases with onset in infancy [36]. In our study, gender distribution was very similar for both sexes (1 M:1F). When only pediatric patients (< 16 years of age) were considered, the distribution showed a higher number of males (62%) than female (36%) as observed by other authors [37, 38]. Syndromes with intellectual disabilities and multiple congenital anomalies were the most represented categories among the undiagnosed conditions. We used the software tool *Phenotyps* to collect and standardize the terms describing the gamut of the clinical features. Phenotype data of undiagnosed persons are commonly reported in a highly variable mode of clinical annotations, ranging from simple phenotype checklists to sizeable paper reports [39]. This might reduce or impede the value of a careful phenotypization necessary for a helpful combination of phenotypic and genomic data. Conversely, deep phenotyping allow a systematic cataloguing of the manifestations of the disease. To use standardised terms from Human Phenotype Ontology (HPO), all running international programs on URDs including our currently adopt web-based applications to structure phenotypic information. It has been shown that precise and detailed phenotypic description by computer based tools also facilitate the interpretation of genomic data speeding up the final diagnosis of infants in the neonatal intensive care units, improving neonatal outcome and inpatients costs [40].

In our study, a total of 13/71 (18,3%) family cases were selected to be characterized by WES, a few cases were familial, leading to a total number of 20 individuals (28,2%) investigated by WES; these led to the diagnosis of 7/13 new identified gene/variants, giving to patients the possibility to have a diagnosis after many years. The low number of patients selected was related to the relatively limited financial support of the project, nonetheless, a molecular diagnostic yield of 53,8% was achieved. This value was rather comparable to the diagnostic rates reported in other studies in these last few years for paediatric and adult undiagnosed conditions (25–50%) [40–44].

Although genomic investigations are conventionally used at end of a long diagnostic process, after standard investigation and single or multi-gene panel sequencing, there are many academic centers where WES is the favourite molecular diagnostic test for people with suspected genetic conditions, giving higher molecular diagnostic yield than traditional molecular tests for patients [45–47]. These results provide strong evidences for an increased diagnostic and clinical utility of WES as a first-line molecular test with suspected monogenic disorders. Stark et al. reported a high diagnostic rate by using genomic analysis together with a clear benefit in clinical management and genetic counselling in a relevant percentage of infants enrolled in their study [30]. Advantages were also referable to health and psychosocial impact, reproductive outcome and likely cost-effectiveness [37]. Nonetheless, the adoption of exome sequencing as first line investigation (as we did in some of our cases) for undiagnosed cases deserves further evaluation among specialists and stakeholders as this attitude could lessen in some circumstance the optimal clinical reasoning of health care in practice and education.

It is of note that many patients who undergo genome sequencing remain undiagnosed; in such circumstances, integration of multiple data types can further improve diagnoses. Above all, an additional clinical characterization on the basis of the results of the genomic analysis has been showed to increase the percentage of diagnosis [44]. Then, functional information from model organisms (Drosophila, Zebra Fish, *Caenorhabditis elegans*, etc.), RNA sequencing (trascriptoma) and data about small molecule metabolites (metaboloma) can enhance diagnostic yield in undiagnosed conditions [35, 39].

Besides the identification of disease genes as diagnostic clues of previously unsolved cases, further aims of the project were to study candidate variants of unknown clinical significance and share data from undiagnosed patients in international platforms/networks. Sharing both -omics and phenotypic data among members of IURDN and UDNI was indeed one of the general principles stated in the program. As indicated by the UDNI board members [10–12], each participant shared a minimum of 5 undiagnosed cases through the international Phenome Central repository for clinicians and scientists working in the RDs community [18–20].

Encouraging international collaboration would have meant allowing clinicians to find matches, additional cases of the same unsolved condition and contact their submitters around the world. Cases were participated in an open and highly visible way respecting at the same time the privacy of the patients profiled. Once users enter their patients’ data, they are connected to other patient profiles within PhenomeCentral sharing similar phenotypes and genotypes. PhenomeCentral provides a user-friendly web interface based on the PhenoTips software, and users can enter de-identified data on their patients also by drawing existing patient records from private PhenoTips installations [20]*.* PhenomeCentral supports the uploading of sequencing data. A patient informed consent for data sharing is required according to data protection regulation and ethical considerations. Each patient record in PhenomeCentral is also accomplished by setting different permissions for global sharing: private (the record is visible only to the owner), matchable (users can discover the existence of matchable cases, but cannot access to the full case without explicit permission from the owner), public (all registered users can view the entered cases). More information on PhenomeCentral are available at: https://www.phenomecentral.org/. In our series, 7 patients out of 12 were found to show potential matches, with different relevance percentage of match similarities with other patients in PhenomeCentral. Although more in-dept comparisons are needed and ongoing, these findings strongly encourage clinicians to participate to specialized international platforms.

## Conclusion

Under-diagnosis and misdiagnosis represent an important burden for people with RDs and their families; national and international collaboration are fundamental to foster and override problems mainly due to uniqueness of patient. The widespread adoption and use of exome sequencing in routine clinical practice for undiagnosed patients is expected to improve diagnostic rates and, if the patient’s phenotype is not suggestive for a specific traditional analysis, reduce test costs, while leading to improvements in patient outcomes and disease management with a renewed emphasis on the discovery of new models of treatment. As programs on how to improve diagnosis of RDs have been implemented over the last years at national and international level, a substantial body of experience has been progressively gained. A series of characteristics that well-organised programs should take into consideration are collaboration and teamwork among experts, education resources for health care professionals to improve their genomics competencies, research training for young investigators, long-lasting funded projects. The goal of these programs is obviously making timely and appropriate diagnoses of the most complex disorders that have escaped from timely medical diagnosis [10, 11]. Additional and undoubting results of such initiatives will be the reduction of the social isolation of the undiagnosed individuals, new management protocols, studies on drug repurposing, supported clinical training, basic and clinical research development, prevention of complications and promotion of global health.

In 2016–2019 period IURDN has grown to include an increasing number of Italian Centres of Expertise thus providing a platform able to share its results both at national and at international level (UDNI). It is hoped that in the future this network will continue to expand its reach to enable many more clinicians and scientists, to utilize its resources to provide answers for undiagnosed patients.

## Data Availability

The datasets generated and analysed during the current study are not publicly available. Phenotypic information of the selected clinical cases are collected and analysed by using the software application “PhenoTips” (https://phenotips.org/). This application was used and customised for the purpose of the project (PhenoTipsISS). Data are available from the corresponding author on reasonable request.

## Abbreviations

RDs: Rare diseases
NIH: National Institutes of Health
OMIM®: Online Mendelian Inheritance in Man
UDP: Undiagnosed Diseases Program
UDNI: Undiagnosed Diseases Network International
NCRDs: National Centre for Rare Diseases
ISS: Istituto Superiore di Sanità
IURDN: Italian Undiagnosed Rare Diseases Network
WES: Whole Exome Sequencing
IC: Informed consents
